# The relationship between health literacy and active and successful aging: a cross-sectional study of active aging service center

**DOI:** 10.1186/s12877-026-08341-6

**Published:** 2026-09-29

**Authors:** Şengül Akdeniz, Karin Wolf-Ostermann

**Affiliations:** 1 Vocational School of Health Services, Department of Health Care Services Elderly Care Program, Akdeniz University, Antalya, 07070 Türkiye; 2 University of Bremen, Institute of Public Health and Nursing Research, PF 330440, Bremen, D-28334 Germany

**Keywords:** Active ageing, Successful ageing, Health literacy, Older adults, Public health

## Abstract

**Background:**

While successful aging focuses on physical health and disease prevention, active aging offers a broader framework encompassing social participation and security. Health literacy is considered a vital tool in achieving these outcomes, yet its specific impact on the synergy between both aging paradigms requires further exploration.

**Objective:**

This study aims to evaluate the relationship between health literacy, successful ageing, and active ageing among older adults and to identify the factors associated with active ageing levels.

**Method:**

This descriptive and cross-sectional study was conducted between October 2024 and May 2025 at the Metropolitan Municipality’s Active Aging Service Center in Antalya, Türkiye. The study included 306 participants aged 65 and over. Data were collected using a Demographic Information Form, the Active Ageing Scale, the Successful Ageing Scale, and the Turkish Health Literacy Scale. Data analysis included descriptive statistics, independent samples t-test, Chi-square, Pearson correlation, and Multiple Linear Regression.

**Results:**

The study involved 306 older adults with an average age of 67.79 ± 3.56 years (79.4% of participants were women, and 70.6% were university graduates). Whilst the majority of participants reported having a chronic illness (66.0%) and taking medication regularly (64.7%), 58.5% engaged in regular physical exercise and 80.7% engaged in regular mental activities. The mean total score for the Active Ageing Scale was 190.10 ± 39.37, for the Successful Ageing Scale it was 57.74 ± 7.61, and for the Health Literacy Index it was 32.97 ± 9.53. Bivariate analyses revealed that higher levels of education, income, regular physical exercise and mental activity were significantly associated with better health literacy and ageing outcomes (*p* < 0.05). Furthermore, positive correlations were found between active ageing, successful ageing and health literacy (*p* < 0.01). Multiple linear regression analysis showed that the model statistically significantly predicted active aging and explained 56.4% of the total variance (R² = 0.564, *p* < 0.05). While age, mental activity, and health literacy emerged as significant predictors, education and regular exercise were found to be insignificant.

**Conclusion:**

Health literacy is a key factor of active and successful ageing. To promote active ageing, older adults care centres should prioritise health literacy.

## Introduction

Globalisation, advances in healthcare and technological developments, coupled with increased life expectancy worldwide, have significantly increased the proportion of older adults in populations, bringing about profound changes in the demographic structure [1, 2]. According to World Health Organisation (WHO) data, the population aged 60 and over is expected to double by 2050, exceeding 2 billion people [2]. With the increase in average life expectancy, the position of older individuals in society and their quality of life are becoming increasingly important. This demographic shift has necessitated that ageing be approached not merely as a biological process, but through the concepts of ‘successful ageing’ and ‘active ageing,’ which involve individuals actively maintaining their physical, mental, and social well-being [3, 4].

Traditional perspectives have generally regarded aging as an inevitable decline and have overlooked the ability of older adults to remain active and contribute to society. In 2002, the WHO introduced the concept of active ageing as as “the process of optimizing opportunities for health, participation and security in order to enhance quality of life as people age” [5]. This approach emphasizes older people’s rights, strengths, and potential, encouraging their continued involvement in family and community life according to their needs and abilities. The term “active” was defined as “continuing participation in social, economic, cultural, spiritual and civic affairs, not just the ability to be physically active or to participate in the labour force” [5]. Active ageing supports physical, social, and psychological well-being, while also reducing (healthcare) costs as a result of remaining healthy and longer in employment while also participating in social life [6]. Based on based on the optimisation of health, lifelong learning, participation and security it has become a key framework for public health policies aimed at improving older adults’ quality of life and addressing global ageing challenges. Even if there is no clearly recognized empirical concept how to measure the construct of active aging, several attempts exist to measure active ageing, linking components of active aging to survival or defining active ageing profiles [7–9]. A review published in 2006 highlighted the lack of a generally recognized definition for ‘successful ageing,’ which led to a critical examination of the construct’s predictors [10]. Over the years, the definition of successful ageing has shifted from a biomedical focus toward more subjective aspects of the ageing process. Successful aging represents the optimal functional status of an older adult [3], while active aging is concerned with the continuity of social and physical participation [5]. In this study, health literacy is positioned as a critical tool that enables older adults to transition from merely ‘surviving’ to ‘actively engaging’ by managing their own health processes, thereby facilitating the attainment of ‘Successful Aging’ status.

Successful ageing encompasses physical, functional, social, and psychological health domains. It also emphasises the integrated management of multidimensional factors such as health literacy, social participation, cognitive stimulating activities, and regular physical activity [10, 11]. Although active and successful aging are sometimes used as synonyms, distinct conceptual differences exist [6]. While successful aging is primarily outcome-oriented (e.g., maintaining health), active aging focuses on the process, emphasizing social rights and opportunities for participation [6]. Therefore, this study includes measures for both constructs to obtain a comprehensive perspective and explore potential conceptual differences.

Health literacy, one of the most fundamental factors affecting independence in old age, encompasses individuals’ ability to access, understand, evaluate, and use basic health information to make informed decisions [12]. Today, older adults’ attitudes toward health are changing, and they are adopting a more proactive approach this makes the role of health literacy in active aging even more critical [13]. The WHO defines health litereacy as “representing the personal knowledge and competencies that accumulate through daily activities, social interactions and across generations. Personal knowledge and competencies are mediated by the organizational structures and availability of resources that enable people to access, understand, appraise, and use information and services in ways that promote and maintain good health and well-being for themselves and those around them.” [14]. Recent studies show that older adults generally have low levels of health literacy, which leads to difficulties in managing chronic diseases and consequently disrupts active ageing processes. Effective interventions to improve older people’s health literacy become important for active and successful aging. Several factors contribute to inadequate health literacy among older populations, including socio-economic status, educational background, and cognitive functioning, as highlighted in recent systematic reviews [15].

A recent systematic review regarding interventions for health literacy in older adults indicates that outcomes are generally more favorable when programs are community-oriented, educational, and focused on specific targets, particularly when integrated with technology [16].

Furthermore, recent evidence emphasizes the impact of targeted health literacy training on older adults’ health outcomes, reinforcing the necessity for structured, evidence-based interventions in clinical and community settings [17].

Recent research indicates a bidirectional predictive relationship between health literacy and successful ageing among older adults [18]. Although the positive association of health literacy with ageing practices has been emphasised in the Turkish literature [18, 19], there are limited studies that examine these variables together and analyse their predictive capacity.

This study is positioned within this framework. Since social support mechanisms and structures that encourage social participation play a critical role in active and successful aging, the Active Aging Service Center implemented by the Antalya Metropolitan Municipality provide important platforms that facilitate both physical and mental stimulation. These centers serve to reduce social isolation among older adults and strengthen their sense of belonging [20, 21]. This study aims to assess health literacy, levels of successful and active aging, and related predisposing factors in a sample of older adults registered at the center in question. The findings will contribute significantly to the development of evidence-based intervention strategies and policies aimed at improving the well-being of the older population.

## Methods

### Study design

This study was designed as a descriptive and correlational survey model to determine the relationship between health literacy, active ageing, and successful ageing levels among older individuals and their respective predisposing factors. The study followed the STROBE guidelines for reporting cross-sectional studies.

### Research population and sample

The study was conducted between October 2024 and May 2025 amongst individuals aged 65 and over who were registered at the Active Ageing Service Centre, which operates under the Directorate of Social Services of Antalya Metropolitan Municipality. The centre provides comprehensive support services to its 1,490 registered members, including sports, psychological counselling, artistic activities and routine health checks.

Taking into account the total population of 1,490, the required minimum sample size was determined to be 316 using the finite population formula, based on a 95 per cent confidence level, a 5 per cent margin of error and a p-value of 0.50. During the participant selection process, members visiting the centre were approached in turn; after accounting for those who did not meet the participation criteria, refused to take part, or withdrew, the research was successfully completed with a final analysis sample of 306 individuals.

### Inclusion and exclusion criteria

The study followed specific inclusion and exclusion criteria to ensure the reliability and feasibility of the sample.

#### Inclusion criteria were

Being aged 65 years or over.

Having actively attended the centre for at least three months.

Meeting the centre’s admission requirements, specifically being capable of independent self-care and free from severe cognitive or psychiatric disorders.

#### Exclusion criteria were

Inability to complete the survey due to cognitive, physical, or communication limitations.

Insufficient time to participate in the study.

Refusal to participate.

### Data collection tools

Data were collected using a structured questionnaire comprising a Demographic Information Form, Active Ageing Scale (AAS), Successful Ageing Scale (SAS), and Turkish Health Literacy Scale-32 (TSOY-32).

#### Socio-demographic and health information form

This form was developed by the authors to collect participants’ personal information. This form includes items that inquire about participants’ personal information such as age, gender, education, income, and marital status, as well as the presence of chronic diseases, medication use, frequency of routine health check-ups, and participation habits in physical/mental activities at the centre. Specifically, the mental activities evaluated within the scope of the centre’s programs included cognitive and educational tasks (such as learning English, chess, and technology use), artistic and handicraft workshops (such as painting, wood painting, stone painting, folk dances, theater, and playing the Turkish frame drum), as well as physical exercise and sports (such as general exercise, dance sports, and table tennis). For this study, regular physical activity is operationally defined as engaging in moderate-intensity physical activity for at least 150 min per week, distributed across at least three days, in line with international guidelines.

#### Active Aging Scale (AAS)

Developed by Rantanen et al. [22] and validated and reliability tested in Turkish by Demir Erbil and Hazer [23], the scale consists of a total of 68 items. The five-point Likert-type scale covers four subscales: Goals, Functional Capacity, Opportunities, and Activities. A higher score on the scale (range 0-272 points) indicates a higher level of active ageing. In the original study, Cronbach’s Alpha coefficient was reported as 0.95.

#### Successful Aging Scale (SAS)

Developed by Reker [24]and adapted into Turkish by Hazer and Özsungur [25], the scale consists of two subscales: Healthy Lifestyle (3 items) and Coping with Problems (7 items), comprising a total of 10 items. A higher total score (range 10–70) on the 7-point Likert-type scale indicates a higher level of successful ageing.

#### Turkish Health Literacy Scale-32 (TSOY-32)

Developed by Okyay and colleagues [26] based on the European Health Literacy Survey (HLS-EU) conceptual framework, the Turkey Health Literacy Scale-32 (TSOY-32) is a 32-item instrument designed to measure the processes of accessing, understanding, evaluating, and applying health-related information. The index score, calculated using five-point Likert-type responses, is standardised to a range of 0–50 using the formula (Index = (Arithmetic Mean − 1) × (50/3). A higher index score indicates a higher level of health literacy (0–25 points: insufficient, 26–33 points: problematic-limited, 34–42 points: adequate, 43–50 points: excellent health literacy). In addition to the total index score, the TSOY-32 encompasses two distinct dimensions: “Treatment and Service” and “Disease Prevention and Health Promotion.” These dimensions are derived from the scale’s subitems and represent participants’ functional capacities regarding the use of clinical services and the proactive management of their health. These subscores are calculated using the same standard formula as the total index, thereby enabling a comparative analysis of participants’ health literacy skills across different health contexts [26].

### Ethical considerations

This study was approved by the Clinical Research Ethics Committee of Akdeniz University (Decision No.: TBAEK-448, Date: July 3, 2024), and written permission was also obtained from the institution where the study was conducted. All procedures were conducted in accordance with the Declaration of Helsinki. Before data collection began, the purpose and nature of the study were explained to all participants, and their voluntary participation was confirmed through verbal and written informed consent, which was a prerequisite for inclusion in the study.

### Statistical analysis

Data analysis was performed using the SPSS (Statistical Package for the Social Sciences) 23.0 software package. Frequency, percentage, arithmetic mean, and standard deviation were used to determine the demographic characteristics of the participants. Data normality was assessed both visually (histograms and Q-Q plots) and through analytical tests (Kolmogorov–Smirnov tests).

For intergroup comparisons, the independent samples t-test was used for two-category variables, and the Chi-square test was used for comparing categorical data. Pearson correlation analysis was applied to determine the direction and strength of the relationship between the scale total scores. Multiple linear regression analysis was performed to determine the factors affecting the level of active aging. The regression model was checked for multicollinearity using the variance inflation factor (VIF) and tolerance values. The statistical significance level was set at *p* < 0.05. P-values in the descriptive analyses were not corrected for multiple testing and will be reported as nominal p-values due to the more explorative nature of this study.

## Results

We included 306 older individuals in our study. The majority of the sample consisted of women (79.4%), nearly two-thirds of the participants were married (63.7%), and living with family (71.6%). The mean age of the participants was 67.79 ± 3.56 years, and the median age was 66 years (range: 65–80 years). Participants had a high education level (university graduates 70.6%) and less than a quarter reported an income less than expenses (22.2%).

Despite a chronic disease rate of 66% and the regular use of medication for chronic illnesses (64.7%), more than half of the participants (58.5%) engage in regular physical exercise and a large proportion (80.7%) engage in regular mental activities. A total of 306 participants took part in training and activities across 12 different disciplines. Current data indicate that the community’s interest in physical activity and language learning is predominant. When the participants’ levels of active aging were examined, it was found that the “functional capacity” dimension had the highest mean score (57.58 ± 9.10), while the “activities” dimension had the lowest mean score (40.87 ± 14.15). The mean total score on the Active Aging Scale was determined to be 190.10 ± 39.37. On the Successful Aging Scale, the ‘coping with problems’ score (40.37 ± 5.41) was found to be higher than the ‘healthy lifestyle’ score (17.37 ± 2.96). Among the subdimensions of health literacy, the score for the “treatment and services” subdimension (34.23 ± 9.17) was higher than the score for the “disease prevention and health promotion” subdimension (31.70 ± 10.60). The overall score for the TSOY-32 Health Literacy Index was 32.97 ± 9.53 (Table 1).

**Table 1:** Distributions of the descriptive characteristics of the participants (*N* = 306)

Descriptive characteristics		*n*	%
Gender	Women	243	79.4
	Men	63	20.6
Marital status	Married	195	63.7
	Single	111	36.3
Living (with)	Alone	87	28.4
	with family	219	71.6
Education status	Illiterate	16	5.2
	Primary School	8	2.6
	Middle School	15	4.9
	High School	51	16.7
	University	216	70.6
Income status	Income exceeds expenses	238	77.8
	Income is less than expenses	68	22.2
Chronic disease	Yes	202	66.0
	No	104	34.0
Regular use of medication	Yes	198	64.7
	No	108	35.3
Regular physical exercıse	Yes	179	58.5
	No	127	41.5
Regular mental activity	Yes	247	80.7
	No	59	19.3
Health check-up frequency	Once a month	7	2.3
	Once every six months	161	52.6
	Once a year	70	22.9
	Longer than one year	68	22.2
Health literacy levels	Inadequate	48	15,7
	Problematic	108	35,3
	Adequate	98	32,0
	Excellent	52	17,0
Types of events attended	Exercise and Sports	79	25.8
	English Language	69	22.5
	Technology	48	15.7
	Chess	29	9.5
	Folk Dances	16	5.2
	Theater	15	4.9
	Painting/Drawing	15	4.9
	Dance Sports	10	3.3
	Wood Painting	10	3.3
	Turkish Frame Drum	7	2.3
	Table Tennis	4	1.3
	Stone Painting	4	1.3

		Mean± (SD)	Median(min-max)
	Age (years)	67.79 ± 3.56	66 (65–80)
Active Aging (AAS)	Goals	46.92 ± 10	47.0 (20.0–68.0)
	Functional capacity	57.58 ± 9.10	59.0 (23.0–68.0)
	Opportunities	44.72 ± 12.33	47.0 (10.0–68.0)
	Activities	40.87 ± 14.15	42.5 (0.0–65.0)
	Total score	190.10 ± 39.37	191 (84–268)
Successful Aging (SAS)	Healthy Lifestyle	17.37 ± 2.96	18.0 (6.0–21.0)
	Coping with Problems	40.37 ± 5.41	42.0 (23.0–49.0)
	Total score	57.74 ± 7.61	60.0 (31.0–70.0)
Health Literacy (TSOY-32)	Treatment and Service	34.23 ± 9.17	33.33(4.17-50.0)
	Disease Prevention and Health Promotion	31.70 ± 10.60	32.29 (3.13-50.)
	Total score	32.97 ± 9.53	32.81 (3.13- 50)

*SD *standard deviation

Table 2 shows the sociodemographic and behavioral factors associated with levels of health literacy. Our findings indicate that a higher level of education, a higher income, and engaging in regular physical exercise and mental activities are statistically significantly associated with higher levels of health literacy (*p* < 0.05). Other factors, such as gender, lifestyle, presence of chronic diseases, frequency of health checkups, and marital status, did not show a statistically significant association with levels of health literacy.

**Table 2:** The relationship between participants’ sociodemographic characteristics and their level of health literacy (*n* = 306)

		Inadequate	Problematic	Adequate	Excellent		
		*n*	*n*	*n*	*n*	X^2^	*p*
Gender	Women	33	87	83	40	5.293^a^	0.152
	Men	15	21	15	12		
Marital status	Married	25	77	63	30	6.324^a^	0.097
	Single	23	31	35	22		
Living	Alone	15	26	33	13	2.819^a^	0.420
	with family	33	82	65	39		
Education status	High School and below	17	22	28	23	10.620^a^	0.014*
	University	31	86	70	29		
Income status	Income equals expenses	29	88	78	43	10.141^a^	0.017*
	Income is less than expenses	19	20	20	9		
Chronic disease	Yes	29	71	69	33	1.668^a^	0.644
	No	19	37	29	19		
Regular use of medication	Yes	21	70	73	34	13.349^a^	0.004*
	No	27	38	25	18		
Regular Physical Exercıse	Yes	23	48	67	41	23.800^a^	< 0.001*
	No	25	60	31	11		
Regular Mental Activity	Yes	34	78	83	52	21.440^a^	< 0.001*
	No	14	30	15	0		
Health check-up frequency	Six months and under	25	60	49	34	3.432^a^	0.330
	One year and above	23	48	49	18		

^a^ Chi-square Test, *p* < 0.05

According to Table 3, significant differences in aging scores were found based on lifestyle and demographic factors. Regular physical exercise and mental activity were identified as the most critical factors; those who engaged in these activities scored significantly higher on all dimensions of both scales (*p* < 0.001). Furthermore, the higher “Coping with Problems” and “Successful Aging” scores of individuals living alone indicate that these individuals have a stronger perceived level of independence.

**Table 3:** Comparison of successful and active aging scale scores according to participant characteristics

	*n*	Healthy Lifestyle (mean ± SD)	Coping with Problems (mean ± SD)	Successful Aging Scale (mean ± SD)	Goals (mean ± SD)	Functional capacity (mean ± SD)	Oppor- tunities (mean ± SD)	Activities (mean ± SD)	AAS (mean ± SD)
Gender	Women (243)	17.56 ± 2.85	40.65 ± 5	58.21 ± 7.19	46.79 ± 10.13	57.74 ± 8.80	45.10 ± 12.52	40.80 ± 14.75	190.43 ± 39.97
	Men (63)	16.65 ± 3.29	39.30 ± 6.49	55.95 ± 8.91	47.41 ± 9.55	57.00 ± 10.29	43.30 ± 11.55	41.16 ± 11.68	188.87 ± 37.23
*p*		**0.030**	0.078	**0.036**	0.663	0.568	0.303	0.857	0.780
Education status	High School and below (90)	16.41 ± 3.38	41.06 ± 5.16	57.47 ± 7.79	49.14 ± 10.90	57.37 ± 9.67	45.48 ± 13.36	42.24 ± 14.12	194.23 ± 43.46
	University (216)	17.77 ± 2.68	40.09 ± 5.50	57,86 ± 7.56	46.00 ± 9.48	57.68 ± 8.88	44.42 ± 11.89	40.30 ± 14.17	188.39 ± 37.51
*p*		< 0.001*	0.145	0.685	**0.012**	0.787	0.515	0.275	0.237
Marital status	Married (195)	17.53 ± 2.77	40.05 ± 5.68	57.57 ± 7.73	45.84 ± 9.87	57.04 ± 8.89	44.24 ± 11.79	41.24 ± 12.35	188.35 ± 37.52
	Single (111)	17.10 ± 3.28	40.95 ± 4.87	58.05 ± 7.44	48.82 ± 9.99	58.55 ± 9.45	45.59 ± 13.24	40.23 ± 16.92	193.19 ± 42.42
*p*		0.224	0.145	0.604	**0.012**	0.163	0.371	0.580	0.302
Living with	Alone (87)	17.29 ± 2.44	41.67 ± 3.88	58.95 ± 5.73	48.98 ± 9.66	56.98 ± 11.25	45.14 ± 13.59	39.86 ± 17.50	190.95 ± 44.03
	with family (219)	17.41 ± 3.16	39.86 ± 5.84	57.26 ± 8.21	46.11 ± 10.04	57.83 ± 8.12	44.57 ± 11.82	41.27 ± 12.61	189.77 ± 37.46
*p*		0.725	**0.002**	**0.042**	**0.022**	0.523	0.731	0.494	0.826
Income status	Income equals expenses (238)	17.32 ± 3.12	39.98 ± 5.58	57.30 ± 7.97	46.55 ± 9.57	57.63 ± 9.38	44.96 ± 11.73	41.92 ± 12.74	191.05 ± 37.99
	Income is less than expenses (68)	17.56 ± 2.35	41.75 ± 4.55	59.31 ± 6.01	48.24 ± 11.37	57.44 ± 8.14	43.91 ± 14.31	37.22 ± 17.92	186.81 ± 44.02
*p*		0.494	**0.008**	**0.026**	0.267	0.874	0.581	**0.047**	0.472
Presence of chronic disease	Yes (202)	17.24 ± 2.90	40.59 ± 4.93	57.83 ± 6.80	47.19 ± 9.61	57.11 ± 9.01	44.47 ± 11.23	40.07 ± 13.55	188.84 ± 37.02
	No (104)	17.63 ± 3.09	39.95 ± 6.24	57.58 ± 9.03	46.40 ± 10.76	58.51 ± 9.27	45.23 ± 14.28	42.43 ± 15.22	192.58 ± 43.66
*p*		0.286	0.330	0.782	0.517	0.203	0.610	0.167	0.432
Regular medication intake	Yes (198)	17.42 ± 2.80	40.59 ± 5.26	58.01 ± 7.17	47.34 ± 9.06	57.34 ± 8.99	45.10 ± 11.32	40.51 ± 14.13	190.28 ± 37.20
	No (108)	17.28 ± 3.26	39.98 ± 5.69	57.26 ± 8.39	46.15 ± 11.54	58.04 ± 9.34	44.06 ± 14.03	41.55 ± 14.26	189.79 ± 43.24
*p*		0.68	0.351	0.411	0.353	0.522	0.509	0.54	0.916
Regular Physical Exercıse	Yes (179)	18.30 ± 2.40	42.32 ± 4.19	60.62 ± 5.75	50.13 ± 9.41	59.54 ± 9.06	47.67 ± 11.67	43.94 ± 14.50	201.27 ± 38.50
	No (127)	16.07 ± 3.20	37.62 ± 5.75	53.69 ± 8.09	42.40 ± 9.05	54.83 ± 8.47	40.58 ± 12.08	36.55 ± 12.50	174.37 ± 35.10
*p*		< 0.001*	< 0.001*	< 0.001*	< 0.001*	< 0.001*	< 0.001*	< 0.001*	< 0.001*
Regular Mental Activity	Yes (247 )	17.87 ± 2.62	41.19 ± 4.94	59.05 ± 6.77	49.00 ± 9.43	58.97 ± 7.89	46.92 ± 11.80	42.57 ± 14.18	197.46 ± 37.37
	No (59 )	15.31 ± 3.42	36.97 ± 5.99	52.27 ± 8.55	38.22 ± 7.20	51.78 ± 11.39	35.58 ± 10.15	33.76 ± 11.75	159.34 ± 32.15
*p*		< 0.001*	< 0.001*	< 0.001*	< 0.001*	< 0.001*	< 0.001*	< 0.001*	< 0.001*
Health check-up frequency	Six months and under (168 )	17.12 ± 3.10	40.16 ± 5.56	57.28 ± 8.01	46.87 ± 10.26	58.23 ± 9.47	45.41 ± 11.53	43.08 ± 13.49	193.59 ± 38.26
	One year and above (138 )	17.68 ± 2.77	40.63 ± 5.23	58.31 ± 7.10	46.99 ± 9.72	56.80 ± 8.62	43.90 ± 13.24	38.19 ± 14.54	185.87 ± 40.42
*p*		0.095	0.448	0.234	0.919	0.167	0.293	**0.003**	0.090

*AAS *Active Aging Scale, *SD *standard deviation

*p < 0.001, *p* < 0.05, Student's t-test

According to Table 4, Pearson correlation results indicate statistically significant, positive relationships among the active aging, successful aging, and health literacy scores (*p* < 0.01). A moderate to strong association was observed between successful aging and active aging (*r* = 0.518), suggesting that these scales measure related concepts. Furthermore, a strong positive relationship was found between health literacy and active aging (*r* = 0.717), indicating that as health literacy improves, the capacity for active aging also increases significantly. Finally, a moderate to strong association was found between successful aging and health literacy (*r* = 0.464).

**Table 4:** Correlations between successful ageing, health literacy, and active ageing scores

Scales	1.	2.	3.
1. Successful Aging Scale	1		
2. Health Literacy	*r* = 0.464**	1	
3. Active Aging Scale	*r* = 0.518**	*r* = 0.717**	1

** Pearson correlation test

*p* < 0.05 (*n* = 306)

As shown in Table 5, the multiple linear regression model was found to be statistically significant in predicting the total active aging score (R² = 0.564, adjusted R² = 0.556, F = 64.550, *p* < 0.005). When individual predictors were examined, health literacy (beta = 0.557, *p* = 0.001), regular mental activity (beta = -0.156, *p* = 0.001), and age (beta = 0.081, *p* = 0.047) made statistically significant contributions; however, educational level (beta = -0.004, *p* = 0.917) and regular exercise (beta = -0.085, *p* = 0.056) were not found to be significant at the *p* < 0.05 level. Since the reference category for the variables “regular mental activity” and “regular exercise” which were included in the analysis as binary (dummy) variables coded as “1 = Yes, 2 = No” was set as “2 = No,” negative B coefficients were obtained. This finding indicates that individuals who engage in these activities regularly (those who answered “Yes”) have active aging scores that are 15.534 and 6.803 points higher, respectively, compared to the reference group, thereby confirming the positive, enhancing effect of mental activity and exercise on active aging.

**Table 5:** Multiple linear regression analysis results for factors predicting active aging level

Variables	B	SE	β	t	*p*	Tolerans	VIF
(Constant)	58.515	34.11	-	1.715	0.087	-	-
Age	0.691	0,347	0.081	1.992	0.047	0.879	1.138
Education level	-0.13	1.242	-0.004	-0.105	0.917	0.948	1.055
Regular Mental Activity	-15.534	4.246	-0.156	-3.658	0.001	0.802	1.247
Regular Exercise	-6.803	3.541	-0.085	-1.921	0.056	0.739	1.352
Health Literacy	2.067	0.162	0.557	12.736	0.001	0.762	1.313

Multiple Linear Regression Analysis

*B *Unstandardized Regression Coefficient, *SE *Standard Error, *β *Beta/ Standardized Regression Coefficient, *VIF *Variance Inflation Factor

*p* <  0.05  ; R2 = 0.564;  Adjusted R2 = 0.556;  F = 64.550

## Discussion

This cross-sectional study, which examines the relationship between health literacy and active and successful aging among older adults attending the Active Aging Service Center, demonstrates that health literacy is a critically associated with active and successful ageing. As shown in Table 5, the regression model which includes the variables age, education level, mental activity, health literacy, and regular exercise was found to be statistically significant in predicting the total active aging score; these independent variables explain 56.4% of the total variance (R² = 0.564, adjusted R² = 0.556, F = 64.550, *p* < 0.05 ). When individual predictor variables were examined, health literacy, regular mental activity, and age were found to make significant contributions to active aging, whereas educational level and regular exercise did not reach statistical significance at the *p* < 0.05 level. In particular, since regular mental activity and regular exercise were dummy-coded as 1 = Yes and 2 = No (with “No” used as the reference category), these variables yielded negative B coefficients (B = -15.534 and B = -6.803, respectively). This indicates that individuals who engage in these activities regularly score higher on active aging compared to the reference group, thereby confirming their positive, developmental effects rather than an inverse relationship.

It is important to note that, given the cross-sectional design of this study, these findings reflect statistical prediction and variance explanation rather than causal relationships; an R^2^ of 0.564 indicates that 56.4% of the observed variance in the active ageing score is explained by the variables included in this regression model, but it does not establish that these variables causally determine active ageing. Although both active and successful aging scales were administered to evaluate participants comprehensively reflecting the broad conceptual framework of our study and its title the multiple linear regression model was specifically constructed using the Active Aging Scale as the primary dependent variable. This methodological choice was primarily driven by the setting of the study, as data were collected directly at Active Aging Service Centers, whose core mission and environment naturally prioritize active aging dynamics.

Among these predictors, age demonstrated a statistically significant positive relationship with the total active aging score. This finding indicates that older individuals in the study group maintained higher levels of active aging; this phenomenon may be attributed to greater psychological resilience, accumulated life experience, or effective coping strategies developed over time, and it aligns with previous studies highlighting the dynamic nature of aging [17, 20, 21, 27]. Recent literature emphasizes that the aging process should be viewed not only as disease management but also as the preservation of internal capacity and functional abilities [27]. Our findings emphasize that successful and active aging cannot be viewed merely as a passive outcome of chronic disease management; rather, it is a dynamic process strongly supported by health literacy and meaningful social participation within a supportive community environment. Within the context of municipal service centers, older adults are provided with an integrated ecosystem where cognitive, physical, and social components intersect, and these factors collectively shape their capacity to successfully manage the aging process.

Our analysis shows that health literacy is the strongest predictor of active aging. We interpret this result as a reflection of the unique community-based context of our study. Unlike clinical settings, where health literacy is typically measured by the ability to follow medical instructions, in the context of the Active Aging Service Center, health literacy emerges as an active decision-making competency. Our participants do not merely receive health information; they actively apply this information to enhance their daily routines, social interactions, and mental engagement. This transition from a “passive patient” to an “active community member” highlights the important role of health literacy as a key contributor to successful ageing in this population group. Therefore, in our study, we argue that the high predictive power of health literacy is directly linked to the integrative nature of municipal services, which foster an environment where health information can be immediately translated into action.

Beyond theoretical understanding, health literacy serves as a fundamental tool for evaluating and applying health information, thereby directly shaping an individual’s quality of life and social integration. This underscores that health literacy is not merely a clinical necessity but an indispensable element for maintaining internal capacity and functional independence in older age [28, 29]. In contrast to factors typically viewed as disadvantages in older populations, the highly educated sample group in our study demonstrates the extent to which older adults with high access to resources can derive maximum benefit from social and cognitive programs; thereby further underscoring the importance of ensuring access to such centers not only for similarly privileged or supported cohorts but also for disadvantaged groups with lower educational levels [28, 29]. Furthermore, although an association between education and health literacy was observed in the bivariate analysis, it is noteworthy that education did not emerge as a statistically significant independent predictor of active aging in the multivariable regression model when adjusted for other covariates.

Our findings highlight the vital role that community-based interventions such as those offered at our Active Aging Center play in promoting active aging. Recent meta-analytic evidence shows that group-based and multi-component health literacy interventions significantly improve not only literacy levels but also self-care, self-efficacy, and quality of life among older adults [17]. The effectiveness of our municipal programs stems from the promotion of social dialogue, participation, and the sharing of life experiences among older adults, as noted in the literature [30–32]. Prior evidence indicates that protective health behaviours, such as cancer screenings, achieve true continuity only when supported by self-efficacy and information management capacity [31]. Furthermore, while mobility limitations are known to trigger social isolation, participation in physical activity effectively integrates older adults into their social environment [32]. Regarding the relationship between physical activity and our measures, older adults who engage in regular physical activity scored statistically significantly higher on both the Active Aging Scale and measures of successful aging (*p* < 0.001). This result supports the premise that physical and social participation are mutually reinforcing components of healthy aging. Although a sedentary lifestyle is often associated with social isolation, our findings show that structured physical activity programs offered by Active Aging Service Centers effectively integrate older adults into their communities. Furthermore, this sustained physical participation appears to be associated with reduced vulnerability to the potential negative consequences of sensory or physical decline on overall social participation.

The fact that mental activity emerged as a significant predictor in our study (*p* < 0.001) is largely related to the structural diversity of the opportunities offered by the municipality’s Active Aging Service Center. In this context, mental activity is not an abstract cognitive exercise but rather a holistic social experience facilitated through a wide variety of activities, such as language courses, chess, and art workshops. In our view, these activities provide the cognitive stimulation necessary to preserve “inner capacity” in later life, which in turn directly fosters the proactive approach essential for active aging. We observe that when older adults engage in meaningful mental activities, their self-confidence in managing their own aging process increases, and this transforms cognitive health into a catalyst for successful aging. This demonstrates that municipal programs serve as facilitators; the diversity of these programs enables older adults to maintain their mental engagement, which in turn raises their Active Aging Scale scores.

The strong positive correlation between health literacy and the Active Aging Scale (*r* = 0.717) suggests that cognitive and behavioral engagement serve as a bridge. Older adults who maintain regular mental and physical routines are likely more adept at navigating complex health information; this, in turn, reinforces their self-efficacy [33, 34]. Interestingly, it has been observed that individuals living alone score higher on the “Coping with Problems” and “Successful Aging” dimensions. This can be interpreted through the lens of independence; individuals with the ability to make autonomous decisions generally perceive their ability to live alone as a personal achievement [35]. In the context of our study, municipal support programs serve as a vital buffer that prevents the social isolation often associated with living alone and enhances life satisfaction by helping older adults maintain their autonomy [36, 37]. Finally, the moderate-to-strong association (*r* = 0.464) between the Successful Aging Scale and health literacy reinforces the notion that internal psychological resources, such as coping mechanisms, are deeply intertwined with an individual’s capacity to access and utilize health-related information. Consequently, it is imperative that local government services prioritize intergenerational health promotion and community-based programs that aim to both improve health literacy and foster social participation in order to reduce health inequalities [38, 39].

### Limitations of the study

This study has certain limitations. Firstly, the sample is limited to older individuals registered at a single Active Aging Service Center in a city centre, which may limit the generalisability of the findings to the entire older adults population in Türkiye. It is important to note that these participants are already engaged in organised lifelong learning and socialisation programmes, which may result in a selection bias. This demographic may possess higher baseline motivation and activity levels than home-bound or non-registered older adults. Therefore, our study population represents older adults who are sufficiently independent and engaged to attend an active aging center, and consequently, these findings may not represent frail, socially isolated, homebound, institutionalized, or less health-literate older adults. Secondly, the cross-sectional design of the study prevents the establishment of a definitive cause-and-effect relationship between variables. Thirdly, the data are based on self-reported measures, which may be subject to recall bias. Notwithstanding the limitations previously mentioned, the utilisation of validated scales and the calculated sample size serve to enhance the reliability of the results.

## Conclusion and recommendations

This study has elucidated the interplay between health literacy which is critically important for individuals to maintain their quality of life amidst a rapidly aging global population and active and successful aging, specifically focusing on older adults attending an Active Aging Center. The findings confirm that aging is not merely a phase of medical decline but a dynamic process shaped by information management, social support, and lifestyle choices. The most fundamental finding is that health literacy is the strongest predictor of active aging. Individuals with a high capacity to understand and evaluate health information can enhance their self-efficacy and successfully navigate aging trajectories. Additionally, the impact of regular physical and mental activities underscores the importance of holistic lifestyle strategies in preventing social isolation.

As a notable finding, the high perception of successful aging among individuals living alone highlights the strong association with community-based support structures, such as active aging centers. These institutions help prevent isolation among older adults living alone and enable them to maintain their independence. However, the lower scores observed in groups with limited education and income emphasize the urgent need for targeted interventions to ensure health equity in later life.

In light of these findings, the following recommendations are proposed:

- Public Health and Intervention Programs: Community-based programs targeting older adults should be designed holistically to integrate health literacy development with active aging strategies.
- Institutional and Local Policies: The number of active aging centers should be expanded, and these facilities should be equipped with educational modules specifically designed to support older adults from diverse socioeconomic backgrounds.
- Combating Sedentary Lifestyles: Initiatives that encourage physical and social participation among older adults should be strengthened to prevent isolation and maintain functional independence.
- Future Research: Longitudinal studies are warranted to investigate the causal mechanisms linking health literacy, community engagement, and successful aging trajectories over time.

## Data Availability

The dataset supporting the conclusions of this article consists of sensitive health and sociodemographic information obtained from older adults registered at the Metropolitan Municipality Active Ageing Centre. The data are protected by national data protection legislation and institutional policies. The ethical approval and informed consent obtained from the participants specifically restrict the sharing of data in public repositories to ensure confidentiality. Consequently, the datasets are not publicly available. However, de-identified data may be made available from the corresponding author upon reasonable request, provided that (i) a formal data use agreement is signed and (ii) necessary permissions are obtained from the relevant ethics committee and the municipal authorities.
